# Supplementary material for: Hyperpolarized nanodiamond with long spin-relaxation times
Source: Nat Commun. 2015 Oct 9;6:8459. doi: 10.1038/ncomms9459 (PMC4633625; doi:10.1038/ncomms9459)
Supplement: Supplementary Information — Supplementary Figures 1-8, Supplementary Tables 1-2, Supplementary Note 1 and Supplementary References [file ncomms9459-s1.pdf]

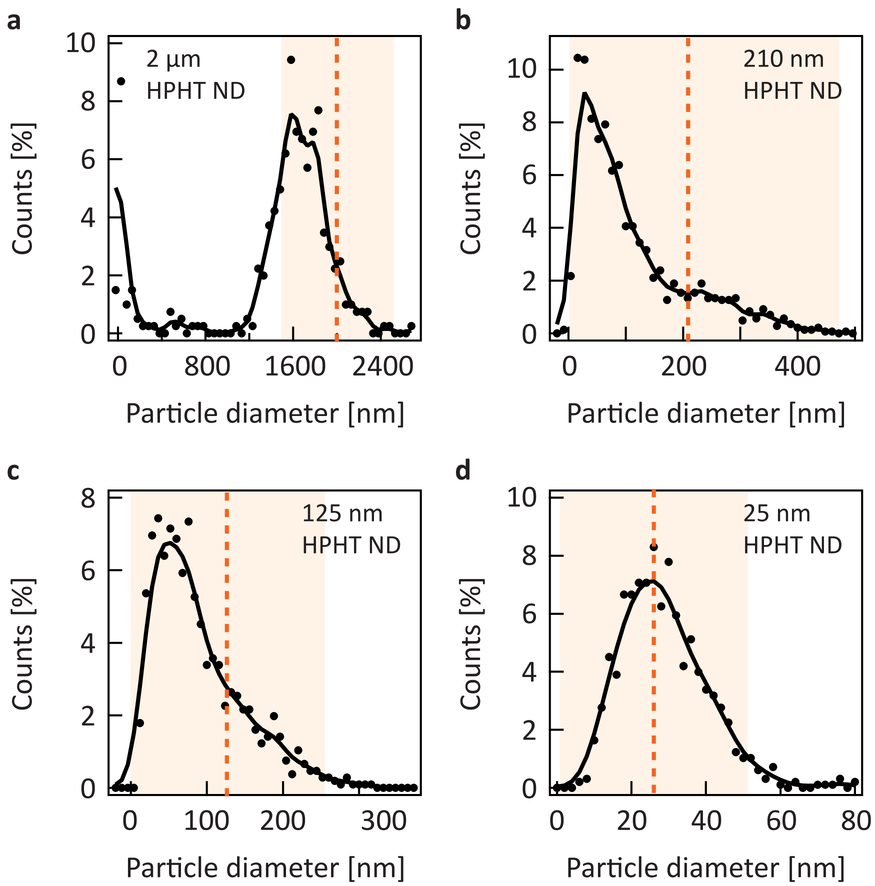

Supplementary Figure 1: Size distribution of ND determined from SEM images. **(a)** 2 µm ND, **(b)** 210 nm ND, **(c)** 125 nm ND, and **(d)** 25 nm ND. ND particle areas are measured using image analysis of SEM images and converted to particle diameters (black dots) assuming spherical particles. The solid line is a guide to the eye. The orange shaded region is the size range, and the dashed orange line is the median particle size as specified by the supplier.

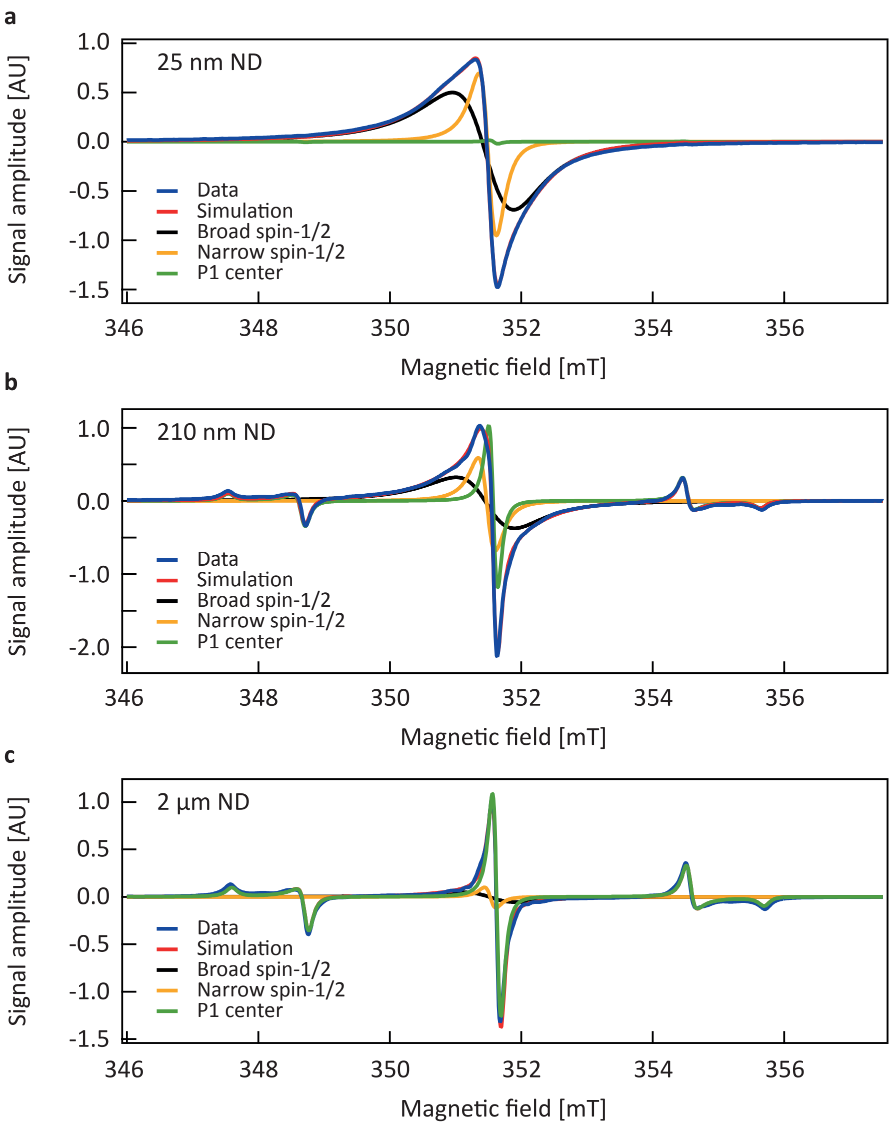

Supplementary Figure 2: ESR spectra of various NDs. **(a)** 25 nm ND **(b)** 210 nm ND, and **(c)** 2 µm ND. Data (blue) is simulated (red) with three components: a broad spin-1/2 component (black) a narrow spin-1/2 component (yellow), and a P1-centre component (green). Fit parameters are linewidths, relative intensities and g-factors. The g-factors stayed constant over all ND sizes, with $g_{\mathrm{broad}} = 2.0027$, $g_{\mathrm{narrow}} = 2.0027$, and $g_{\mathrm{P1-centre}} = 2.0021$.

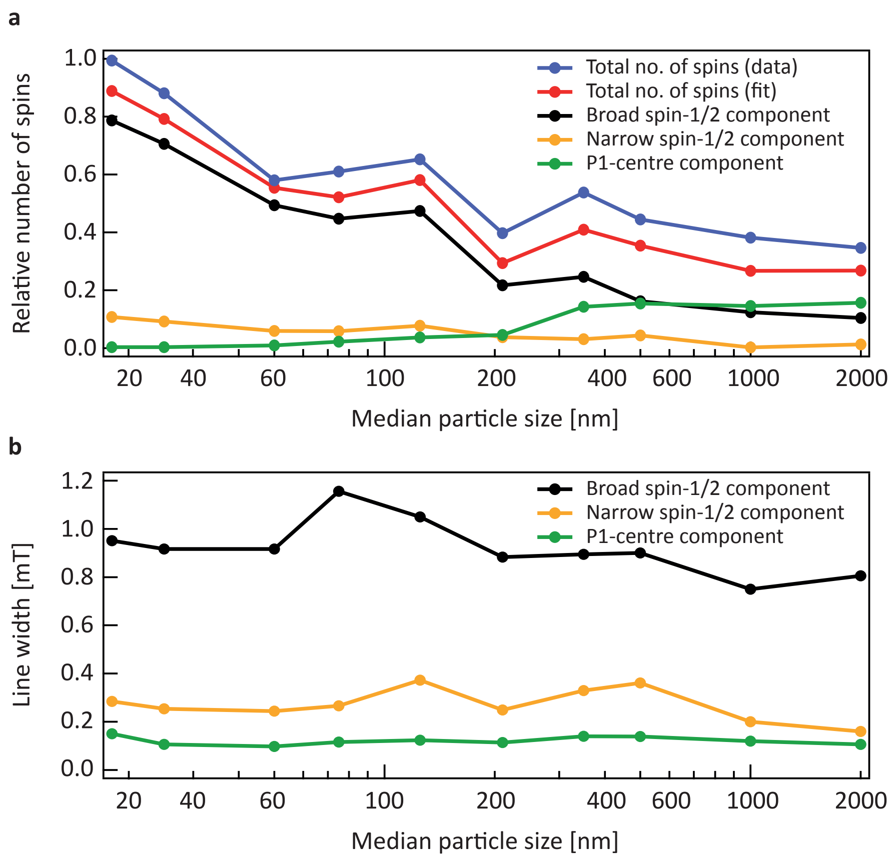

Supplementary Figure 3: ESR fit results of various NDs. (a) Relative number of spins in ND as a function of ND particle size. Individual components (data in blue, simulation in red, broad spin-1/2 component in black, narrow spin-1/2 component in yellow and P1-centre in green) were integrated to determine the number of spins. The total number of spins decreases with increasing particle size. The same behavior is seen in the broad component and narrow component. In contrast the number of P1-centres increases as ND particle size increases. Data has been normalized to the highest electron signal (18 nm ND). **(b)** Linewidths of the fitted components: broad spin-1/2 component in black, narrow spin-1/2 component in yellow, and P1-center in green. The linewidths stay constant over the measured particle size range.

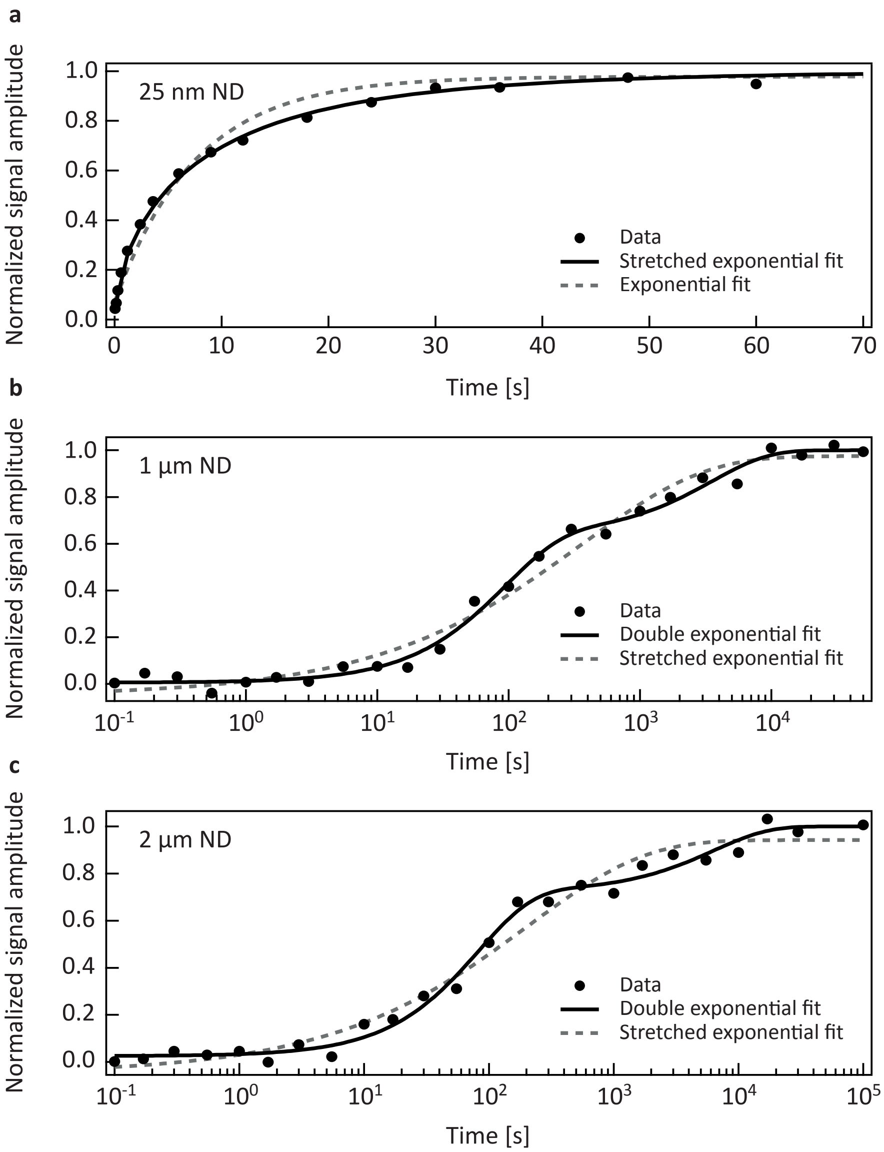

Supplementary Figure 4: T_1 magnetization build up in ND measured at $B = 7$ T. (a) Comparison between a stretched exponential fit (solid black line) and exponential fit (grey dashed line) to the saturation recovery build up data (black dots) of 25 nm ND. The stretched exponential is a better fit for small NDs. **(b, c)** Comparison between a double exponential fit (solid black line) and a stretched exponential fit (grey dashed line) for **(b)** 1 µm ND and **(c)** 2 µm. For larger ND particles the double exponential fit has a smaller variation from the data than a stretched exponential fit.

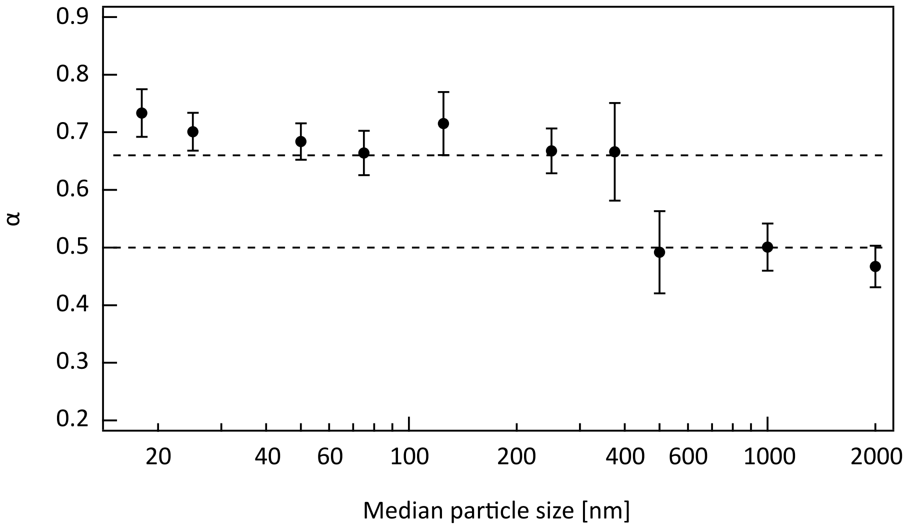

Supplementary Figure 5: α fit parameter for various sized NDs. Stretched exponential component (α) extracted from the best fit to $M = M_0(1 - \exp(-(t/T_1)^{\alpha}))$ of T_1 relaxation data of various sized NDs. Small NDs exhibit a build up with $\alpha = 2/3$ (corresponding to an inhomogeneous distribution of spins). Stretched exponential fits to larger NDs have stretched exponential components at $\alpha = 1/2$ (corresponding to impurity mediated relaxation from a homogenous dirstribution of spins). The error bars indicate the standard deviation of the three measurements that were taken.

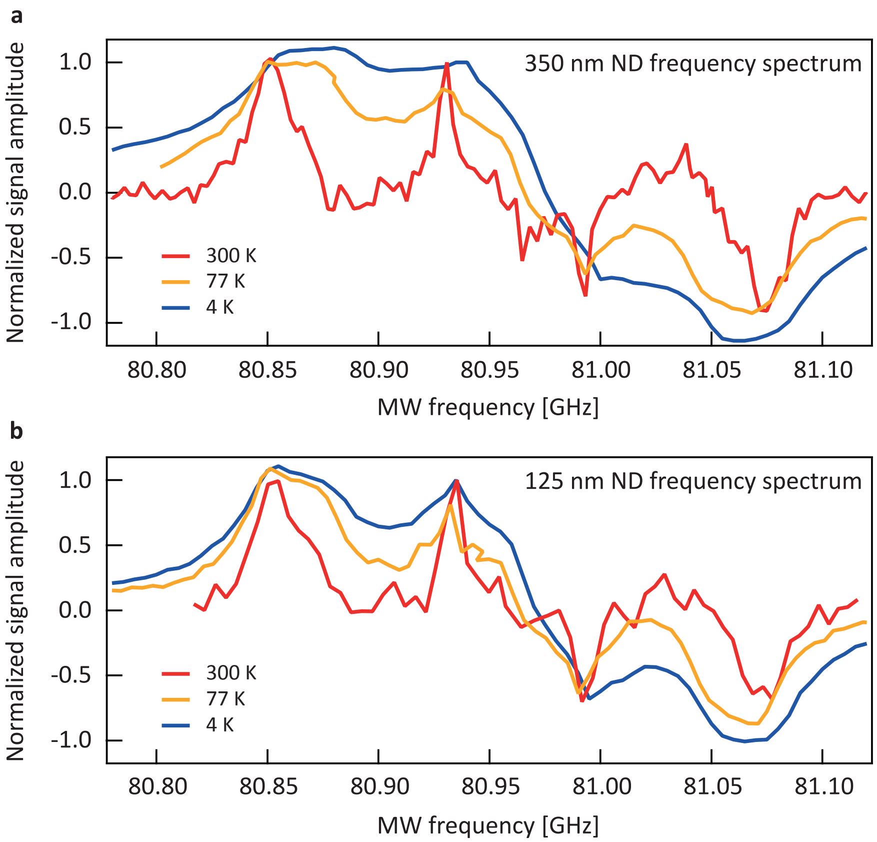

Supplementary Figure 6: Hyperpolarized signal as a function of polarization frequency at various temperatures. Hyperpolarized spectra of **(a)** 350 nm ND, and **(b)** 125 nm ND at $T = 300$ K (red), 77 K (yellow), and 4 K (blue). The traces have been normalized to 1. The features appear at the same frequencies for all NDs examined. The peaks broaden with decreasing temperature, and broaden as particle size increases.

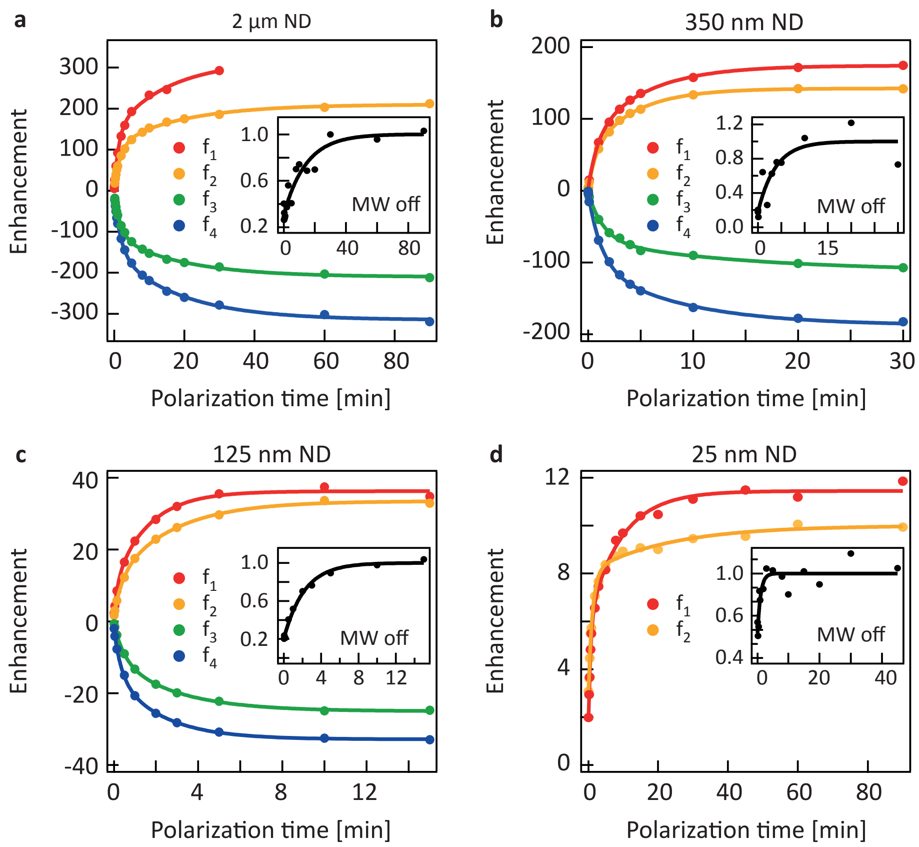

Supplementary Figure 7: Hyperpolarization build up in ND at T = 4 K. Enhancement as a function of polarization time, normalized to the saturated magnetization taken with no microwave power (insets) in ND. Polarization build up was measured at the four frequencies f_1 (red), f_2 (yellow), f_3 (green), and f_4 (blue). Data is shown for **(a)** 2 µm **(b)** 350 nm ND **(c)** 125 nm ND, and **(d)** 25 nm ND. Data is shown in dots and fits to the data are shown in solid lines.

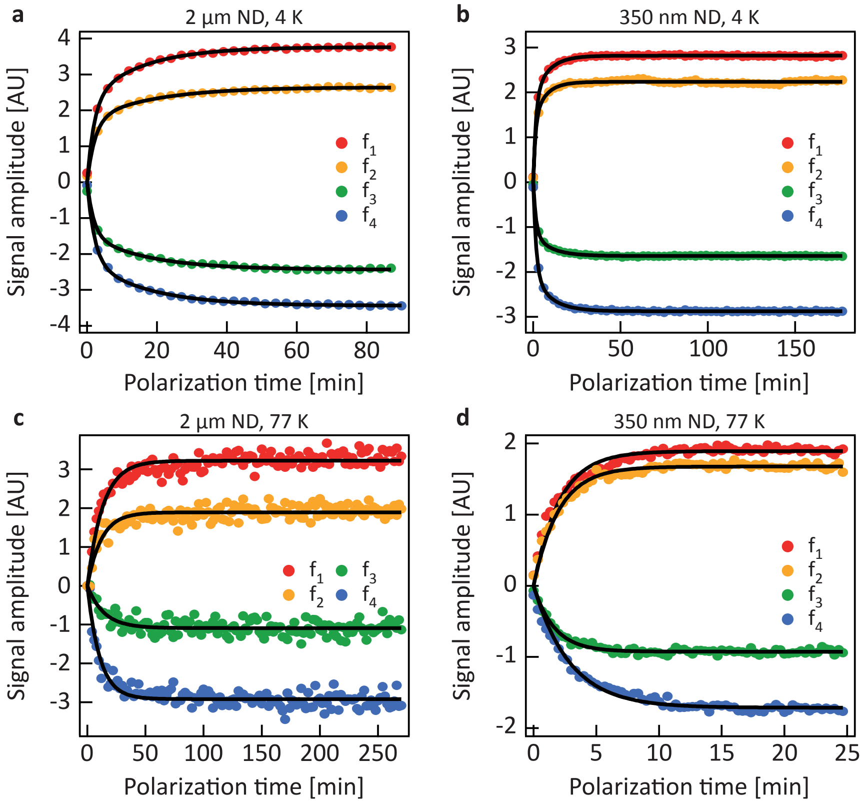

Supplementary Figure 8: Small tip angle hyperpolarization build up in ND at $\boldsymbol{T = 4}$ K and 77 K. Signal amplitude as a function of polarization time in **(a)** 2 µm ND at $T = 4$ K, **(b)** 350 nm ND at $T = 4$ K, **(c)** 2 µm ND at $T = 77$ K, and **(d)** 350 nm ND at $T = 77$ K. Polarization build up was measured at the four frequencies f_1 (red), f_2 (yellow), f_3 (green), and f_4 (blue). Data is shown in dots and solid lines are fits to $M = M_0 \cos(\theta)^{n-1}(A_1 e^{-(n-1)TR/T_{1\text{long}}} + A_2 e^{-(n-1)TR/T_{1\text{short}}})$ at $T = 4$ K, and $M = M_0 \cos(\theta)^{n-1} e^{-(n-1)TR/T_1}$ at $T = 77$ K.

Supplementary Table 1: Hyperpolarization build up in ND using saturation recovery.

Particle size	Frequency [GHz]	$T_{1,\text{short}}$ [min]	$T_{1,\text{long}}$ [min]
2 µm ND	$f_1 = 80.880$	1.3	16
	$f_2 = 80.940$	1.9	19
	$f_3 = 81.000$	1.9	19
	$f_4 = 81.060$	2.0	19
	MW off	$T_1 = 14$	
350 nm ND	$f_1 = 80.870$	0.8	5.2
	$f_2 = 80.940$	0.7	4.2
	$f_3 = 81.000$	1.4	14
	$f_4 = 81.060$	1.0	6.0
	MW off	$T_1 = 4$	
125 nm ND	$f_1 = 80.880$	0.2	1.6
	$f_2 = 80.945$	0.4	2.8
	$f_3 = 81.005$	0.48	2.8
	$f_4 = 81.075$	0.34	2.2
	MW off	$T_1 = 2.2$	
25 nm ND	$f_1 = 80.880$	0.6	9
	$f_2 = 80.955$	1.1	22
	MW off	$T_1 = 1.0$	

Summary of the fit parameters for hyperpolarization build up in ND at $T = 4$ K using a saturation recovery pulse sequence.

Supplementary Table 2: Hyperpolarization build up in ND using a small tip angle pulse sequence.

Particle size	Experimental parameters	Frequency [GHz]	$T_{1,\text{short}}$ [min]	$T_{1,\text{long}}$ [min]
2 µm ND	$T = 4$ K	$f_1 = 80.880$	2.0	16
	$\theta = 9°$	$f_2 = 80.940$	2.3	20
	$TR = 3$ min	$f_3 = 81.000$	1.9	18
		$f_4 = 81.060$	2.1	18
350 nm ND	$T = 4$ K	$f_1 = 80.870$	1.5	8.4
	$\theta = 9°$	$f_2 = 80.940$	1.3	7
	$TR = 3$ min	$f_3 = 81.000$	1.6	10.2
		$f_4 = 81.060$	1.7	10.2

Particle size	Experimental parameters	Frequency [GHz]	T_1 [min]
2 µm ND	$T = 77$ K	$f_1 = 80.880$	15.2
	$\theta = 11.25°$	$f_2 = 80.930$	13.5
	$TR = 2$ min	$f_3 = 80.990$	16.5
		$f_4 = 81.075$	12.0
350 nm ND	$T = 77$ K	$f_1 = 80.860$	2.1
	$\theta = 4.5°$	$f_2 = 80.930$	2.0
	$TR = 20$ s	$f_3 = 80.993$	1.7
		$f_4 = 81.064$	3.0

Summary of the fit parameters for hyperpolarization build up in ND using a small tip angle pulse sequence.

Supplementary Note 1:

Feasibility of imaging with hyperpolarized nanodiamonds

We expect to perform hyperpolarized ND imaging in a preclinical scanner with a small tip angle 2D fast spin echo (FSE) sequence, with ^{1}H-^{13}C co-registration. Overlaying ^{13}C images of functionalized nanodiamonds on high resolution ^{1}H anatomical images would provide information in a similar format to those seen in PET/MRI[1]. Here, we present calculations estimating a pixel signal-to-noise ratio (SNR) of $\sim$ 11 for a nanodiamond concentration of $1\,\mathrm{mg\,mL^{-1}}$. This SNR value is for $2\,\mathrm{mm} \times 2\,\mathrm{mm}$ sized pixels in a 5 mm slice assuming significant polarization loss during transfer from polarizing cryostat to imager. We note that nanodiamond concentrations of 2 mg mL^{-1} have previously been used in vivo[2].

First, we consider the fundamental limit to MRI resolution set by the observed transverse coherence time T_2^*, which is reached when the frequency line-width of the signal is approximately equal to the frequency separation between each pixel[3]:

$$dz \sim \frac{1}{\gamma G \pi T_2^*} \qquad \text{(Supplementary Equation 1)}$$

where dz is the pixel length, γ the nuclear gyromagnetic ratio and G is the peak gradient strength. Our nanodiamond samples have $T_2^* \sim 250\,\mu\mathrm{s}$, which corresponds to a fundamental resolution limit of 0.25 mm in a preclinical scanner or 2.5 mm in a whole body MRI scanner (assuming typical peak gradient strengths for these systems of $G = 500\,\mathrm{mT\,m^{-1}}$ and $G = 50\,\mathrm{mT\,m^{-1}}$ respectively).

Next, we consider the pixel SNR that would be possible from our hyperpolarized nanodiamond samples in an imaging experiment. Our 2 µm ND samples have a polarization after DNP at 4 K, P_{DNP}, of $\sim$ 8%. The thermal polarization, P_{thermal}, at $B_0 = 7$ T and $T = 300\,\mathrm{K}$, is 0.0006%, as given by the Boltzmann distribution:

$$P = \frac{h\gamma B_0}{2k_{\mathrm{B}}T} \qquad \text{(Supplementary Equation 2)}$$

Where γ is the gyromagnetic ratio and k_{B} is Boltzmann's constant. The free induction decay (FID), after a $\pi/2$ pulse, from a 0.1 g, thermally polarized, 2 µm ND sample was acquired in our 7 T spectroscopic probe under the matched filter condition, $t_{\mathrm{acq}}/T_2^* = \pi/2$,

where t_{acq} is the acquisition time. The Fourier transform of this FID has $\mathrm{SNR}_{\pi/2\text{-thermal}} = 35$. Preliminary transfer measurements between the hyperpolarizer and 7 T detection magnet have shown a sample transfer efficiency, η, of 10%. Hence, we predict that, after sample transfer of a hyperpolarized sample to our 7 T spectrometer, we will have an SNR post transfer, $\mathrm{SNR}_{\pi/2\text{-PT}}$, of 45 000,

$$\mathrm{SNR}_{\pi/2\text{-PT}} = \eta\, \mathrm{SNR}_{\pi/2\text{-thermal}}\, P_{\mathrm{DNP}}/P_{\mathrm{thermal}} \qquad \text{(Supplementary Equation 3)}$$

Scaling this SNR value to give an expected sensitivity in a preclinical imaging experiment is inherently nontrivial due to the difficultly of estimating noise associated with coil resistance and losses arising from the sample[4–6]. Here we make an SNR estimate for a preclinical scanner on the assumption that our SNR is limited primarily by coil resistance, which is generally true at $B = 7$ T for mouse coils[7].

The SNR of a pickup coil scales as:

$$\mathrm{SNR} \propto \frac{B_{\mathrm{r}}}{I_{\mathrm{r}}} \frac{1}{\sqrt{4k_{\mathrm{B}}TR_{\mathrm{c}}}} \qquad \text{(Supplementary Equation 4)}$$

where $\frac{B_{\mathrm{r}}}{I_{\mathrm{r}}}$ is the magnetic field strength of the pickup coil per unit current, T is the coil temperature and R_{c} is the coil resistance. $\frac{B_{\mathrm{r}}}{I_{\mathrm{r}}}$ at the centre of an optimised saddle coil is given by:

$$\frac{B_{\mathrm{r}}}{I_{\mathrm{r}}} = \frac{\sqrt{3}\mu_0 N}{\pi d} \frac{l}{\sqrt{l^2 + d^2}} \qquad \text{(Supplementary Equation 5)}$$

where N is the number of turns in the coil, μ_0 is the permeability of free space, l is the coil length and d is its diameter[8]. If all power dissipation occurs in the coil, then we can estimate the coil resistance from

$$Q = \frac{\omega_0 L_{\mathrm{c}}}{R_{\mathrm{c}}} \qquad \text{(Supplementary Equation 6)}$$

where L_{c} is the coil inductance and ω_0 is the resonance frequency. Typically Q is $\sim$100 at 75 MHz for preclinical imaging and spectroscopic NMR probes. Assuming a homogeneous field across the saddle coil, we estimate from Faraday's law that the coil's inductance scales approximately as[8,9]

$$I_{\mathrm{c}} \sim \frac{Nl^2}{\sqrt{l^2 + d^2}} \qquad \text{(Supplementary Equation 7)}$$

Assuming a 1 turn 40 mm diameter, 60 mm long saddle coil is used for mouse imaging, the ratio $\frac{B_r}{I_r}$ is reduced by 95% compared to the 2 turn 6 mm diameter, 13 mm long coil in our spectroscopic NMR probe. Therefore, the expected SNR after DNP and transfer to the imager is $\mathrm{SNR}_{\pi/2\text{-imager}} = 2100$. This result is very similar to that obtained when the resistance is simply scaled by the ratio of the wire lengths in the coils.

We envision using a small tip angle 2D FSE sequence, similar to that used in Ref. 10, to image ^{13}C. When using a CPMG sequence, our nanodiamond samples have demonstrated T_2 values of approximately 100 ms, which would then allow for many echoes, making a FSE sequence feasible. Based on previous calculations for a 2D gradient-recalled echo (GRE) sequence, we estimate the pixel SNR of a 2D FSE imaging sequence to be[11,12]

$$\mathrm{SNR}_{\text{pixel}} = \frac{N}{N_0^2}\left(\mathrm{SNR}_{\pi/2\text{-imager}}\right)\sin\theta \qquad \text{(Supplementary Equation 8)}$$

Where N is the number of pixels across an $N \times N$ image, N_0 is the number of pixels across the object, θ is the tip angle (setting the acquisition time $t_{\text{acq}} \sim T_2^*$). If the 0.1 g of nanodiamond powder in our sample is uniformly distributed through a $40\,\text{mm} \times 40\,\text{mm} \times 5\,\text{mm}$ phantom there is a nanodiamond concentration of $16\,\text{mg}\,\text{mL}^{-1}$. For a 32×32 pixel image with 2 mm $\times$ 2 mm resolution and 5 mm slice thickness, $N = 32$, $N_0 = 20$. For tip angles 10° and 90°, this gives $\mathrm{SNR}_{\text{pixel}} = 30$ and $\mathrm{SNR}_{\text{pixel}} = 170$ respectively. Normalizing this value, we predict $\mathrm{SNR}_{\text{pixel}} = 11$ at $1\,\text{mg}\,\text{mL}^{-1}$ for a 90° tip angle.

Hence, we estimate that there will be sufficient SNR for hyperpolarized nanodiamond imaging. In practice, the measured SNR will deviate from these values depending on the actual sensitivity of the detection coil and polarization lost during sample transfer. We have also not considered the loss of spin coherence due to T_2 effects during the acquisition sequence. These effects will cause some degradation of the SNR at higher spatial frequencies. A range of linewidth narrowing sequences developed for solid imaging may also help to improve image quality[3].

Supplementary References

[1] Pichler, B. J., Judenhofer, M. S. & Wehrl, H. F. PET/MRI hybrid imaging: devices and initial results. *Eur. Radiol.* **18**, 1077–1086 (2008)

[2] Chow, E. K., *et al.* Nanodiamond therapeutic delivery agents mediate enhanced chemoresistant tumor treatment. *Sci. Transl. Med.* **3**, 73ra21 (2011)

[3] Strange, J. H. & Halse, M. R. Imaging techniques for solids and quasi-solids. *eMagRes*, DOI: 10.1002/9780470034590.emrstm0226 (2007)

[4] Hoult, D. I., & Lauterbur, P. C. The sensitivity of the zeugmatographic experiment involving human samples. *J. Magn. Reson.* **34**, 425–433 (1979)

[5] Gadian, D. G. & Robinson F. N. H. Radiofrequency losses in NMR experiments on electrically conducting samples. *J. Magn. Reson.* **34**, 449–455 (1979)

[6] Parra-Robles, J., Cross, A. R. & Santyr, G. E. Theoretical signal-to-noise ratio and spatial resolution dependence on the magnetic field strength for hyperpolarized noble gas magnetic resonance imaging of human lungs. *Med. Phys.* **32**, 221–229 (2005)

[7] Doty, F. D., Entzminger, G., Kulkarni, J., Kranti, P. & Staab, J. P. Radio frequency coil technology for small-animal MRI. *NMR Biomed.* **20**, 304–325 (2007)

[8] Mispelter, J., Lupa, M. & Briguet, A. *NMR probeheads for biophysical and biomedical experiments* (Imperial College Press, 2006).

[9] Rainey, J. K., DeVries, J. S. & Sykes, B. D. Estimation and measurement of flat or solenoidal coil inductance for radiofrequency NMR coil design. *J. Magn. Reson.* **187**, 27–37 (2007)

[10] Cassidy, M. C., Chan, H. R., Ross, B. D., Bhattacharya, P. K. & Marcus, C. M. In vivo magnetic resonance imaging of hyperpolarized silicon particles. *Nature Nanotech.* **8**, 363–368 (2013)

[11] Tsai, L. L., Mair, R. W., Rosen, M. S., Patz, S. & Walsworth, R. L. An open-access, very-low-field MRI system for posture-dependent ^{3}He human lung imaging. *J. Magn. Reson.* **193**, 274–285 (2008)

[12] Callaghan, P. T. *Principles of nuclear magnetic resonance microscopy* (Oxford University Press, 1993).
